# Laparoscopic cholecystectomy in situs inversus totalis: Case report with review of techniques

**DOI:** 10.1016/j.ijscr.2019.05.050

**Published:** 2019-05-31

**Authors:** Omar AlKhlaiwy, Ahmed Mohammed AlMuhsin, Eman Zakarneh, Mohamed Yassin Taha

**Affiliations:** Department of General Surgery, King Fahd Military Medical Complex, Dhahran, Saudi Arabia

**Keywords:** Situs inversus totalis, Laparoscopic cholecystectomy, Case report

## Abstract

**Introduction:**

Situs inversus totalis (SIT) is a congenital disorder in which the visceral organs are mirrored from their normal anatomical position. Diagnosis and management of cholelithiasis in patient with SIT poses a challenge due to the underlying anatomical variation.

**Presentation of case:**

We report a case of a 40-year-old male patient who presented with an intermittent history of epigastric and left upper quadrant pain for one month. Clinical assessment and radiological investigations confirmed the presence of cholelithiasis with evidence of SIT. The patient underwent elective laparoscopic cholecystectomy with no complication and he had an uneventful recovery. Various intraoperative modification has been made to overcome the technical difficulties encountered due to the underlying anatomical variation.

**Discussion:**

Since the first successful laparoscopic cholecystectomy in patient with SIT performed in 1991, 85 cases have been reporsted in the literature. Surgeons managed to overcome the technical difficulties by adopting various modification in the techniques compared to the conventional laparoscopic cholecystectomy.

**Conclusion:**

The anatomical variation in SIT can influence the localization of symptoms in patient with cholelithiasis leading to a delay in diagnosis and management. Laparoscopic cholecystectomy can be safely performed in these cases. However, it is considered technically challenging procedure and often requires alteration in the technique.

## Highlights

• SIT is a rare congenital anatomy with mirror image transposition of the viscera.
• The underlying anatomical variation poses a challenge in the diagnosis and management of cholelithiasis In patient with SIT.
• Laparoscopic cholecystectomy can be safely performed in these cases.
• It is considered technically challenging procedure and often requires alteration in the technique compared to the conventional laparoscopic cholecystectomy.

## 1 Introduction

Situs inversus (SI) is rare congenital disorder with a an autosomal recessive mode of inheritance [1]. The estimated incidence varies from 1 in 5000 to 20,000 live births [1]. Situs inversus refers to spectrum of transposition of the body viscera, which can be complete (totalis) where both the thoracic and abdominal organs are reversed resulting in mirror image of the normal anatomical structures, or it may be partial (partialis) where either thoracic or abdominal organs are reversed [2]. Whereas the presence of an abnormally positioned organ known as situs ambiguous [3]. Dextrocardia refers to right sided heart which also can be found as different isolated entity [3]. Situs inversus (SI) is associated with various congenital anomalies including congenital heart diseases, renal dysplasia, and biliary atresia [2]. Kartagener's syndrome represents a triad of situs inversus totalis (SIT), bronchiectasis, and sinusitis [2]. Diagnosis and management of symptomatic gallbladder stones in patient with SIT is challenging. Minimal invasive surgery is the preferred option however it carries within many technical difficulties due to the anatomical variation. We present a case of symptomatic gallbladder stone in a patient with situs inversus totalis requiring laparoscopic cholecystectomy (LC), discussing its feasibility and reviewing the surgical techniques in the literature. the work has been reported in line with the SCARE criteria [4].

## 2 Case report

**Fig. 1:**
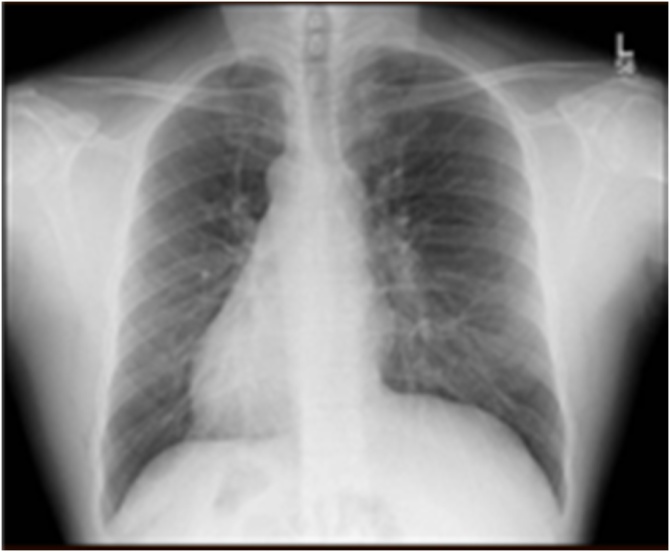
Chest X Ray showing dextrocardia with stomach fundic gas shadow on Right side.

**Fig. 2:**
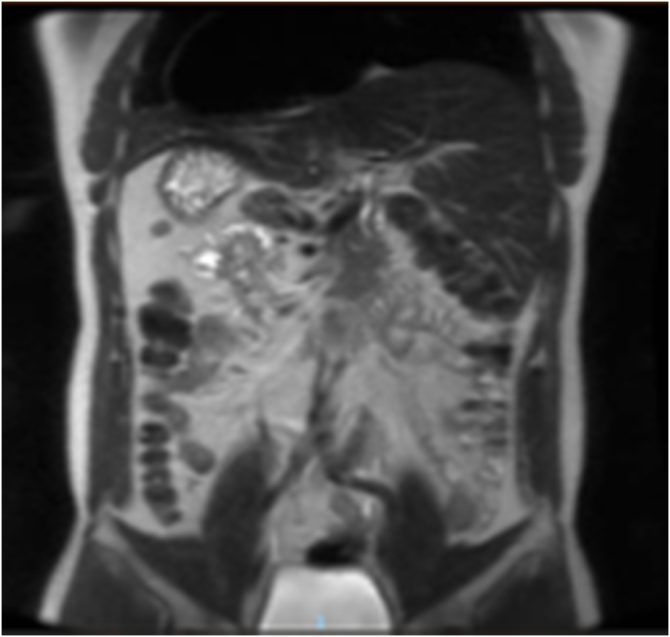
Magnetic Resonance Cholangiopancreatography showing the liver on the left side, and confirming the presence of situs inversus totalis (Coronal View).

**Fig. 3:**
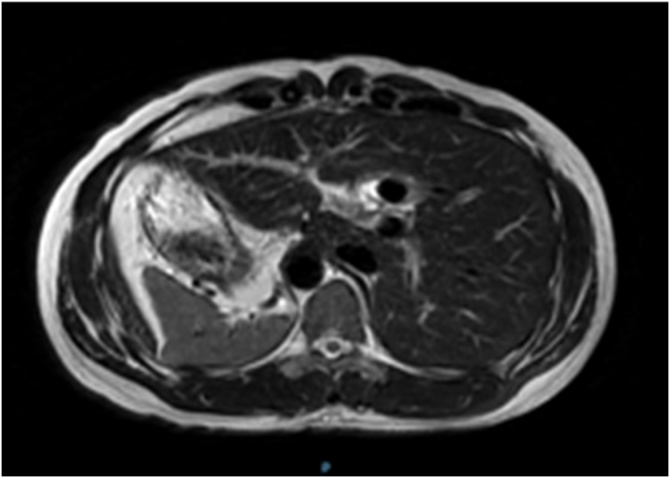
Magnetic Resonance Cholangiopancreatography showing the liver on the left side, and confirming the presence of situs inversus totalis. (Axial View).

A 40-year-old male not known to have any chronic medical illness, presented complaining of epigastric and left upper quadrant pain for 1 month, associated with intermittent nausea and vomiting, and aggravated by fatty meals, with no other associated symptoms. He had frequent visits to the emergency department where he was managed with analgesia and antacids with mild symptomatic improvement. Clinical examination was unremarkable with no evidence of jaundice or abdominal tenderness. His blood test results showed a normal complete blood count, kidney function, and liver function. Chest X Ray revealed dextrocardia with stomach fundic gas shadow on Right side (Fig. 1). Abdominal ultrasonography revealed transpositioning of the solid organs with a left sided liver and gallbladder with a solitary stone and mild wall thickening. We elected to perform a Magnetic Resonance Cholangiopancreatography to delineate the anatomy and to rule out any anomalies within the biliary tree. It confirmed the previously noted findings, showed no evident anomaly within the biliary tree, and confirmed the diagnosis of situs inversus totalis (Fig. 2, Fig. 3). The patient was Scheduled for an elective laparoscopic cholecystectomy.

**Fig. 4:**
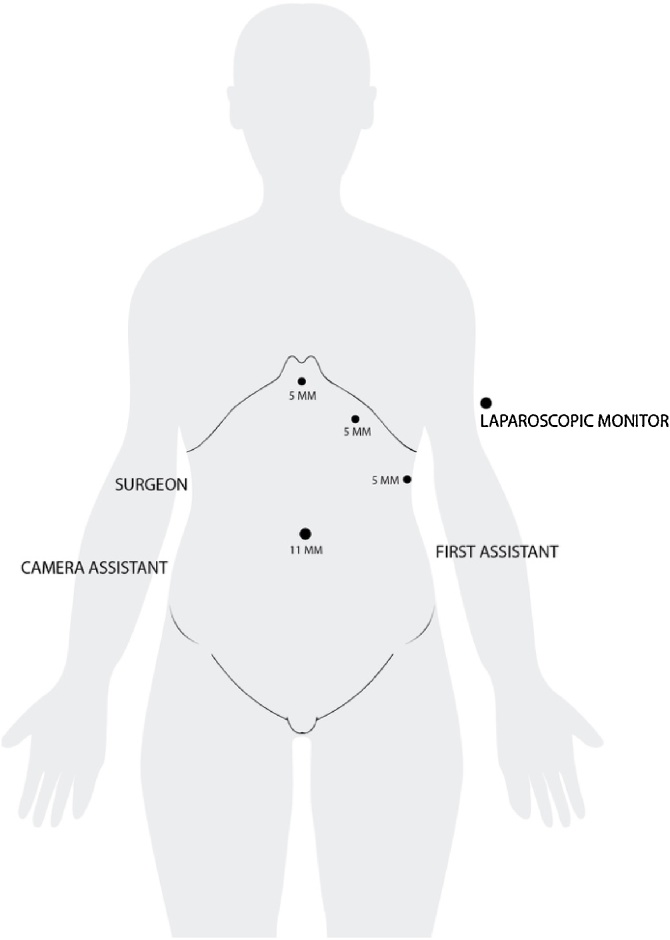
Illustration of the theater setup and port placement.

**Fig. 5:**
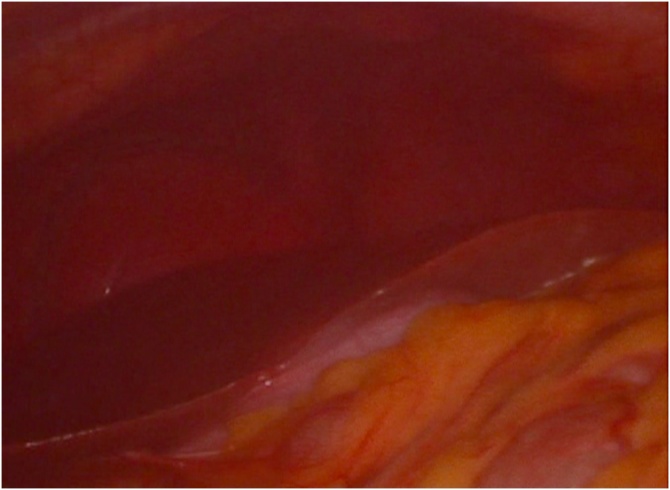
Laparoscopic view showing the position of the liver and the gallbladder in the left side.

The Operating room equipment arrangement was adjusted as Mirror Image of Routine Laparoscopic cholecystectomy (Fig. 4). The Monitor was placed on left side of the patient. The surgeon with the camera assistant were on right side of the patient and the first assistant was on left side of the patient. The abdomen was scrubbed and draped in the standard aseptic technique. The first infraumbilical 11 mm trocar introduced and pneumoperitoneum induced using the open technique. Three 5 mm trocars were placed, at the xiphisternum which was used for the surgeon’s left hand, at the left midclavicular line 2 cm below the costal margin which was used as working port for the surgeon’s right hand and at left anterior axillary line 5 cm from the costal margin which was used for retraction of the gallbladder fundus by the second assistant, respectively. Inspection of the abdominal cavity confirmed the presence of situs inversus totalis, with the liver and the gallbladder positioned in the left side (Fig. 5). The Calot’s triangle was identified. The peritoneum overlying the gallbladder infundibulum was then incised and the cystic duct and cystic artery identified and circumferentially dissected, till the critical view was obtained. The cystic duct and cystic artery were then doubly clipped and divided, through the subcostal port using the right hand. The gallbladder was dissected from its peritoneal attachments using electrocautery and was retrieved using Endoscopic bag through the infraumbilical port. The total operative duration was 80 min, which was longer than the conventional laparoscopic cholecystectomy performed in patient without underlying anatomical variation. It can be attributed to the modification in the technique required to adjust to the mirror image anatomy.

The patient had an uneventful postoperative course and was discharged on postoperative day 1. Pathological examination of the gallbladder confirmed the presence of gallstones with chronic cholecystitis. No postoperative complications were noted during his follow up in the outpatient department.

## 3 Discussion

Situs inversus totalis (SIT) is a rare autosomal recessive congenital anomaly, with a global prevalence of 0.01% [1,5]. It is characterized by the transposition of both thoracic and abdominal viscera resulting in perfect mirror image of their normal anatomical position [5]. It can be associate with various congenital anomalies, such as Kartagener’s syndrome which comprises a triad of SIT, sinusitis and bronchiectasis, and Yoshikawa’s syndrome that is characterized by the presence of SIT, bilateral renal dysplasia, pancreatic fibrosis and meconium ileus [6].

Diagnosis of biliary colic in patient with SIT is challenging due to the underlying anatomical anomaly. They often have an unusual presentation in form of left upper quadrant or epigastric pain, leading to a delay in the diagnosis and management especially in those who are not known to have SIT, as in the reported case. However, there is no evidence suggest that patients with SIT are more susceptible to cholelithiasis [7].

Open Cholecystectomy was the mainstay of management of cholelithiasis in the prelaparoscopic era. Around 40 cases of open cholecystectomy in patients with SIT were reported in the literature [8]. With advancement in surgical techniques, minimal invasive surgery has been introduced. The first case of Laparoscopic cholecystectomy was successfully performed by mouret in 1987, and since then it has become the gold standard approach [9]. In 1991 Campos and sipes performed the first successful laparoscopic cholecystectomy in patient with SIT [10]. Since then 91 cases have been reported in the literature (Table 1). None of these cases reported any complication or have been converted to open cholecystectomy. Thus, it is considered a safe procedure, and not contraindicated in SIT [11,9]. However, it is carries within technical challenges due to the underlying mirror image anatomy which demands meticulous dissection of the biliary tree to avoid iatrogenic injuries [12]. Various techniques have been advocated to overcome these difficulties.

**Table 1:** Summary of reported cases of laparoscopic cholecystectomy in patients with situs inversus totalis.

Serial no. of cases in each series	Author	Year of publication	Diagnosis	Gender	Age
1	Campos and Sipes et al.	1991	Chronic Cholecystitis	Female	39
2	Takei et al.	1992	Cholelithiasis	Female	51
3	Lipschutz et al.	1992	Cholangitis/ CBD calculi	Male	80
4	Goh et al.	1992	Empyema	Male	62
5	Drover et al.	1992	Chronic Cholecystitis	Female	29
6	Huang et al.	1992	Chronic Cholecystitis	Male	36
7	Schiffino et al.	1993	Chronic Cholecystitis	Female	53
8	Mc Dermott and Caushaj et al.	1994	Cholangitis/ CBD calculi	Male	66
9	Malataniet al.	1996	Acute Cholecystitis	Female	25
10	Crosher et al.	1996	Cholelithiasis	Male	63
11	D’Agata and Boncompagni et al.	1997	Chronic Cholecystitis	Female	72
12	Habib et al.	1998	cholecystectomy	Female	45
13/14	Demetriades et al.	1999	Acute Cholecystitis /Chronic Cholecystitis	Female	61/37
15	Djohan et al.	2000	Chronic Cholecystitis /appendectomy	Female	20
16	Wonget al.	2001	Chronic Cholecystitis /CBD calculi	Female	68
17	Dorthi et al.	2001	Chronic Cholecystitis	Female	43
18	Nursal et al.	2001	Chronic Cholecystitis	Female	42
19/20	Yaghan et al.	2001	Chronic Cholecystitis /Chronic Cholecystitis	Female	48/38
21	Al Jumaily and Hoche et al.	2001	Chronic Cholecystitis	Female	46
22	Singh and Dhi et al.	2002	Chronic Cholecystitis	–	–
23	Trongue et al.	2002	Chronic Cholecystitis	Female	28
24	Polychronidis et al.	2002	Chronic Cholecystitis	Male	68
25/26	Oms and Badia et al.	2003	Acute Cholecystitis	Female/ Male	70/65
27	Jesudason et al.	2004	Chronic Cholecystitis	–	–
28	Kang and Han et al.	2004	Chronic Cholecystitis /CBD calculi	Female	64
29	Docimo et al.	2004	Chronic Cholecystitis	Female	41
30	Pitiakoudis et al.	2005	Chronic Cholecystitis	Female	47
31	McKayand Blake et al.	2005	Acute Cholecystitis	Female	32
32	Kamitani et al.	2005	Chronic Cholecystitis	Male	76
33	Puglisi et al.	2006	Chronic Cholecystitis	Female	43
34	Bedioui et al.	2006	Chronic Cholecystitis	Female	58
35	Aydin et al.	2006	Chronic Cholecystitis	Male	35
36	Machado and Chopra et al.	2006	Chronic Cholecystitis	Female	65
37	Kumar and Fusai wt al.	2007	Chronic Cholecystitis	Female	57
38	Fernandes et al.	2008	Chronic Cholecystitis	Female	43
39	Hamdi and Abu hamdan wt al.	2008	Acute Cholecystitis	Male	41
40	Pavlidis et al.	2008	Acute Cholecystitis	Female	34
41	Taskin et al.	2009	Chronic Cholecystitis /Gastric banding	Female	20
42	Masood et al.	2009	Chronic Cholecystitis	Female	42
43	Pereira-Graterol et al.	2009	Chronic Cholecystitis	Female	70
44	Romano et al	2009	Cholelithiasis	Female	67
45	Eisenberg D et al	2009	Cholelithiasis	Male	61
46	Pataki et al.	2010	Chronic Cholecystitis	Female	68
47	Hall et al.	2010	Chronic Cholecystitis	Male	53
48	Gonzalez Valverde et al.	2010	Chronic Cholecystitis	Female	46
49	Sanduc and Toma et al.	2010	Chronic Cholecystitis	Female	64
50/51/52/53/ 54/55	Patle NM et al.	2010	5 Cholelithiasis/ 1 Acute Cholecystitis	5 Female/ 1 Male	36/43/ 27/48/59/33
56	Han et al.	2011	Chronic Cholecystitis	Male	45
57	Ozsoy et al.	2011	Chronic Cholecystitis	Female	65
58	Uludag et al.	2011	Cholelithiasis	Male	49
59	Borgaonkar et al.	2011	Cholelithiasis/ Appendicitis	Female	47
60	Seo KW et al.	2011	Cholelithiasis / Gastric cancer	Male	60
61	Evoli LP et al.	2011	Cholelithiasis	Female	48
62	Iusco el al.	2012	Cholelithiasis	Female	52
63	Elbeshry et al.	2012	Cholelithiasis	Female	24
64	Lochman et al.	2012	Acute Cholecystitis	Female	75
65/66	Demiryilmaz et al.	2012	Cholelithiasis/ Choledocholelithiasis	Female/ Male	55 /51
67	de Campos Martins, Marcus Vinicius Dantas et al.	2012	Cholelithiasis	Female	59
68	Pahwa, Harvinder Singh et al.	2012	Cholelithiasis	Female	46
69	Bozkurt et al.	2012	Cholelithiasis	Male	49
70	Salama et al.	2013	Cholelithiasis	Male	10
71	Arya et al.	2013	Cholelithiasis	Female	35
72	Ali MS et al.	2013	Cholelithiasis	Female	43
73	Khiangte et al.	2013	Cholelithiasis	Male	65
74	Raghuveer et al.	2014	Cholelithiasis	Male	55
75	Reddy et al.	2014	Acute Cholecystitis/ Choledocholelithiasis	Female	45
76	Fang el al.	2015	Gallbladder polyp / Rectal cancer	Female	39
77	Deguchi et al.	2015	Cholelithiasis	Male	66
78	Rosen H et al.	2015	Acute Cholecystitis	Male	36
79	Phothong et al.	2015	Cholelithiasis	Female	39
80	Alsabek et al.	2016	Cholelithiasis	Female	50
81/82/83	Zeeshan et al.	2016	Acute Cholecystitis/ Cholelithiasis / Cholelithiasis	Female	46/44/33
84	Jian-jun et al.	2017	Chronic Cholecystitis	Female	36
85	Rungsakulkij and Tangtawee et al.	2017	Biliary pancreatitis	Male	32
86	Fanshawe and Qurashi et al.	2017	Biliary pancreatitis	Female	53
87	Alam and Santra et al.	2017	Cholelithiasis	Female	20
88	El Hajj et al	2017	Cholangitis/ biliary pancreatitis	Male	61
89	Ying et al.	2017	Cholangitis/ Acute Cholecystitis	Female	51
90	Yogesh et al.	2018	Cholelithiasis / CBD calculi	Female	50
91	Jhobta RS et al.	2018	Cholelithiasis	Female	23
92	Reported Case	2018	Cholelithiasis	Male	40

^*^Only case of Laparoscopic cholecystectomy in SIT were included * Only English articles were included. *CBD: Common bile duct.

In the current literature, the most frequently adopted technique is the four port technique with placement of the laparoscopic equipment, positioning of the surgical team, and ports sites are a mirror image of the standards used in the usual cases [13,14]. The surgeon stands on the right side of the patient along with the camera assistant, and the first assistant stands on the left side. Left-handed instruments are used to grasp Hartmann’s pouch through the subxiphoid port, and the right hand is used for dissection through the left midclavicular subcostal ports [14,15]. Modification of this technique have been reported in the literature, where the assistant retracts the gallbladder infundibulum while the surgeon perform the dissection through the epigastric port with the right hand [8,13]. Some authors adopted a complete mirror image approach by using the left hand for dissection through the subxiphoid port, which could be more suitable option for a left handed or ambidextrous surgeon [16]. Another alternative for the surgeon to be positioned between the patient’s leg while the patient is in Lloyd-Davis position [17]. Recently a laparoendoscopic single-site surgery technique have been reported, which had the advantages of easier dissection with the right hand and better cosmetic result [[18], [19], [20], [21]].

No technique has been considered yet as a standard for such cases. Surgeons should choose any suitable approach taking in account meticulous dissection and critical view achievement before clipping the cystic duct and artery. Intraoperative cholangiogram can be performed in such cases to visualize the anatomy and avoid iatrogenic injury [22]. Rungsakulkij et al. used fluorescent cholangiography by administration of indocyanine green to delineate the extrahepatic biliary tree anatomy [14].

## 4 Conclusion

SIT is a rare congenital anatomy with mirror image transposition of the viscera. This anatomical variation can influence the localization of symptoms in patient with cholelithiasis leading to a delay in diagnosis and management. Laparoscopic cholecystectomy can be safely performed in these cases. However, it is considered technically challenging procedure and often requires alteration in the technique compared to the conventional laparoscopic cholecystectomy.

## Conflicts of interest

The authors declare that there is no conflict of interest regarding the publication of this paper.

## Funding

This case report had no funding or sponsors.

## Ethical approval

This case report is exempt from ethical approval by our institution.

## Consent

Written informed consent was obtained from the patient for publication of this case report and accompanying images. A copy of the written consent is available for review by the Editor-in-Chief of this journal on request.

## Author contribution

OA assisted in the surgery, drafted the manuscript and reviewed the literature. AM wrote the manuscript and reviewed the literature. EZ performed the surgery and reviewed the manuscript. MT supervised the management of the patient and reviewed the manuscript. All authors read and approved the final manuscript.

## Registration of research studies

None.

## Guarantor

Dr. Ahmed Mohammed Al.Muhsin.

## Provenance and peer review

Not commissioned, externally peer-reviewed.

## References

[1] Ren Jian-jun, Li Shu-dong, Geng Ya-jun, Xiao Rui (2017). Modified laparoscopic cholecystectomy technique for treatment of situs inversus totalis: a case report. J. Int. Med. Res..

[2] Ali M.S., Attash S.M. (2013). Laparoscopic cholecystectomy in a patient with situs inversus totalis: case report with review of literature. BMJ Case Rep..

[3] Rosen H., Petrosyan M., Mason R.J. (2015). Cholecystitis in situs inversus totalis. Radiol. Case Rep..

[4] Agha R.A., Borrelli M.R., Farwana R., Koshy K., Fowler A., Orgill D.P., For the SCARE Group (2018). The SCARE 2018 statement: updating consensus surgical CAse REport (SCARE) guidelines. Int. J. Surg..

[5] Fanshawe Angela E.E., Qurashi Kamran (2017). Laparoscopic cholecystectomy for gallstone pancreatitis in a patient with situs inversus totalis. J. Surg. Case Rep..

[6] Demetriades H., Botsios D., Dervenis C., Evagelou J., Agelopoulos S., Dadoukis J. (1999). Laparoscopic cholecystectomy in two patients with symptomatic cholelithiasis and situs inversus totalis. Dig. Surg..

[7] Takei H.T., Maxwell J.G., Clancy T.V., Tinsley E.A. (1992). Laparoscopic cholecystectomy in situs inversus totalis. J. Laparoendosc. Surg..

[8] Lochman P., Hoffmann P., Kočí J. (2012). Elective laparoscopic cholecystectomy in a 75-year-old woman with situs viscerum inversus totalis. Wideochir. Inne /Tech. Maloinwazyjne.

[9] Borgaonkar V.D., Deshpande S.S., Kulkarni V.V. (2011). Laparoscopic cholecystectomy and appendicectomy in situs inversus totalis: a case report and review of literature. J. Minim. Access Surg..

[10] Campos L., Sipes E. (1991). Laparoscopic cholecystectomy in a 39-year-old female with situs inversus. J. Laparoendosc. Surg..

[11] Schiffino L., Mouro J., Levard H., Dubois F. (1993). Cholecystectomy via laparoscopy in situs inversus totalis: a case report and review of the literature. Minerva Chir..

[12] Yaghan R.J., Gharaibeh K.I., Hammori S. (2001). Feasibility of laparoscopic cholecystectomy in situs inversus. J. Laparoendosc. Adv. Surg. Tech. Part A.

[13] Arya S.V., Das A., Singh S., Kalwaniya D.S., Sharma A., Thukral B.B. (2013). Technical difficulties and its remedies in laparoscopic cholecystectomy in situs inversus totalis: a rare case report. Int. J. Surg. Case Rep..

[14] Rungsakulkij Narongsak, Tangtawee Pongsatorn (2017). Fluorescence cholangiography during laparoscopic cholecystectomy in a patient with situs inversus totalis: a case report and literature review. BMC Surg..

[15] Pahwa Harvinder Singh, Kumar Awanish, Srivastava Rohit (2012). Laparoscopic cholecystectomy in situs inversus: points of technique. BMJ Case Rep..

[16] Eisenberg D. (2009). Cholecystectomy in situs inversus totalis: a laparoscopic approach. Int. Med. Case Rep. J..

[17] Patle N.M., Tantia O., Sas mal P.K., Khanna S., Sen B. (2010). Laparoscopic cholecystectomy in situs inversus-our experience of 6 cases. Indian J. Surg..

[18] de Campos Martins M.V., Pantaleão Falcão J.L., Skinovsky J., de Faria G.M. (2012). Single-port cholecystectomy in a patient with situs inversus totalis presenting with cholelithiasis: a case report. J. Med. Case Rep..

[19] Bozkurt S., Coskun H., Atak T., Kadioglu H. (2012). Single incision laparoscopic cholecystectomy in situs inversus totalis. J. Surg. Tech. Case Rep..

[20] Ozsoy M., Haskaraca M.F., Terzioglu A. (2011). Single incision laparoscopic cholecystectomy (SILS) for a patient with situs inversus totalis. BMJ Case Rep..

[21] Han H.J., Choi S.B., Kim C.Y., Kim W.B., Song T.J., Choi S.Y. (2011). Single-incision multiport laparoscopic cholecystectomy for a patient with situs inversus totalis: report of a case. Surg. Today.

[22] Phothong Natthawut, Akaraviputh Thawatchai, Chinswangwatanakul Vitoon, Trakarnsanga Atthaphorn (2015). Simplified technique of laparoscopic cholecystectomy in a patient with situs inversus: a case report and review of techniques. BMC Surg..

